# The complete chloroplast genome sequence of *Moringa oleifera* Lam. (Moringaceae)

**DOI:** 10.1080/23802359.2019.1627922

**Published:** 2019-11-18

**Authors:** Wen Lin, Seping Dai, Yile Chen, Yubin Zhou, Xiaojuan Liu

**Affiliations:** a Guangzhou Institute of Forestry and Landscape Architecture, Guangzhou, Guangdong, China;; b The Affiliated Taihe Experimental School, South China Normal University, Guangzhou, Guangdong, China;; c State Key Laboratory of Bio-Control, Guangdong Provincial Key Laboratory of Plant Resources, School of Life Science, Sun Yat-sen University, Guangzhou, Guangdong, China;; d Landscaping Company of Guangzhou, Guangzhou, Guangdong, China

**Keywords:** *Moringa oleifera*, complete chloroplast genome, automated assembly, phylogenetic analysis

## Abstract

The plant family Moringaceae contains only one genus, *Moringa*, and *Moringa oleifera* is widely cultivated for its young seed pods and leaves used as vegetables and for traditional herbal medicine. In this study, we report the complete chloroplast genome of *M. oleifera*, assembled from whole-genome high-throughput sequencing reads, as a resource for future studies on the phylogeny and evolution of Moringaceae. The chloroplast genome was 160,600 bp in length, with a large single-copy (LSC) region of 88,577 bp, a small single-copy (SSC) region of 18,883 bp, separated by two inverted repeat (IR) regions of 26,570 bp each. It was predicted to contain 131 genes, with an overall GC content of 36.78%. Phylogenetic analysis of 71 protein-coding sequences of 13 plant plastomes showed that *M. oleifera* is closest to *Carica papaya*.

The plant family Moringaceae contains only one genus, *Moringa*, and this genus comprises 14 species (Ramachandram et al. 1980). *Moringa oleifera* Lam. is native to the southern foothills of the Himalayas in northern India. It is now widely grown in more than 30 tropical and subtropical countries and regions in Asia, Africa, and America (Lu 2003; Chen 2005). It is cultivated for its young seed pods and leaves used as vegetables and for traditional herbal medicine. In this study, we characterized the complete chloroplast genome sequence of *M. oleifera* as a resource for future studies on the phylogeny and evolution of Moringaceae.

The fresh leaves of an individual of *M. oleifera* were collected from Germplasm resources nursery of ornamental plants in Guangzhou, China. (The geospatial coordinates is 23°13′56′′N, 113°20′8′′E); and the specimen is stored in the same place. After DNA extraction, a genomic DNA library with an insertion size of 350 bp was constructed, and high-throughput DNA sequencing (paired-end 150 bp) was performed on an Illumina Hiseq X Ten platform (Illumina, San Diego, CA), generating approximately 5 Gb of sequence data. Using an *M. oleifera* rbcL gene sequence (GenBank Accession No. KY697379) as the seed, the chloroplast genome was assembled from the Illumina reads using the program NOVOPlasty (Dierckxsens et al. 2017). Annotation of the chloroplast genome was performed using the Dual Organellar GenoMe Annotator (DOGMA) online tool (Wyman et al. 2004) and Geneious version 10.1 (http://www.geneious.com, Kearse et al. 2012), then manually verified and corrected by comparison with sequences of related species in GenBank.

The complete chloroplast genome sequence of *M. oleifera* (GenBank accession MH939149) obtained in this study was 160,600 bp in length, with a large single-copy (LSC) region of 88,577 bp, a small single-copy (SSC) region of 18,883 bp, separated by two inverted repeat (IR) regions of 26,570 bp each. It was predicted to contain 131 genes, including 87 protein-coding genes, 36 tRNA genes, and 8 rRNA genes. The overall GC content was 36.78%.

To investigate the phylogenetic position of *M. oleifera* Lam., phylogenetic analysis was conducted based on the homologous blocks of chloroplast genome sequences of *M. oleifera* and 12 other species from *Akaniaceae*, *Caricaceae*, *Capparaceae*, *Cleomaceae*, and *Brassicaceae*, with *Arabidopsis arenosa* used as an outgroup, using HomBlocks tool (Bi et al. 2018). The sequences were aligned using MAFFT version 7.307 (Katoh and Standley 2013). A maximum-likelihood tree was then constructed using RAxML (Stamatakis 2014). As shown in Figure 1, *M. oleifera* appears to be phylogenetically closest to *Carica papaya*.

**Figure 1.**
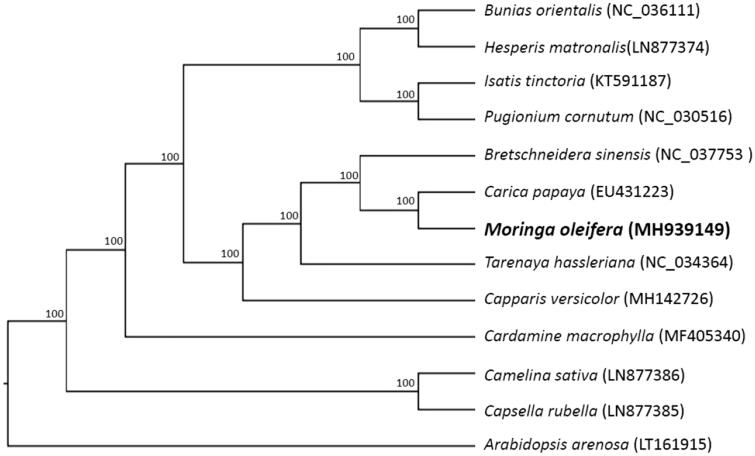
Maximum-likelihood tree based on 13 complete chloroplast genomes, with *Arabidopsis arenosa* as out group. Bootstrap support values (based on 1000 replicates) are shown next to the nodes.
